# Programmed Death Ligand 1 (PD-L1) Tumor Expression Is Associated with a Better Prognosis and Diabetic Disease in Triple Negative Breast Cancer Patients

**DOI:** 10.3390/ijms18020459

**Published:** 2017-02-21

**Authors:** Gerardo Botti, Francesca Collina, Giosuè Scognamiglio, Federica Rao, Valentina Peluso, Rossella De Cecio, Michela Piezzo, Gabriella Landi, Michelino De Laurentiis, Monica Cantile, Maurizio Di Bonito

**Affiliations:** 1 Pathology Unit, Istituto Nazionale Tumori Fondazione “G. Pascale”, via Mariano Semmola, 80131 Napoli, Italy; g.botti@istitutotumori.na.it (G.B.); francescacollina84@gmail.com (F.C.); giosco80@gmail.com (G.S.); federica.rao@gmail.com (F.R.); valent.peluso@gmail.com (V.P.); r.dececio@istitutotumori.na.it (R.D.C.); mauriziodibonito@libero.it (M.D.B.); 2 Department of Breast Surgery and Cancer Prevention, Istituto Nazionale Tumori Fondazione “G. Pascale”, via Mariano Semmola, 80131 Napoli, Italy; m.piezzo@breastunit.org (M.P.); g.landi@istitutotumori.na.it (G.L.); m.delaurentiis@istitutotumori.na.it (M.D.L.)

**Keywords:** TNBC, PD-L1, diabetes

## Abstract

Triple Negative Breast Cancers (TNBC) subtype is an aggressive disease with poor clinical outcome. The only treatment available is surgery followed by chemotherapy or radiotherapy. Programmed death-ligand 1 (PD-L1) is a trans-membrane protein expressed on a wide variety of cells including immune cells, epithelial and vascular endothelial cells. Recently, PD-1/PD-L1 pathway signaling was described as an adaptive immune resistance mechanism enacted by the tumor cells to evade the immune response. Its presence on tumor cell membranes, acquired for this reason, through time, is an important prognostic value. However, data available in the literature about PD-L1 immunohistochemical expression in breast cancer are often discordant and not uniform, probably for the use of different antibodies clones and the high molecular heterogeneity of the different tumor types. The absence of target therapies, in particular for TNBC, has shifted the clinical attention mainly on the role of PD-L1 in this subtype of breast cancer. In this study, we evaluated tumor and TIL (tumor infiltrating lymphocytes) PDL-1 expression in a series of TNBC, included in Tissue Micro Arrays (TMAs), to define its real prognostic value, optimizing immunohistochemistry method with an “approved for diagnostic assay” antibody. PD-L1 expression directly correlated with proliferation index (Ki-67), glycemia, the presence of diabetes and indirectly with menopausal status, presence of lymph node metastasis and relapse. The analysis of Kaplan–Meier showed that an increased PD-L1 expression was strongly associated with better disease-free survival (DFS) but not correlated with overall survival (OS). Our data confirmed that PD-L1 could be an important marker for prognostic stratification and for planning immune checkpoint inhibitors therapies in patients with TNBC.

## 1. Introduction

TNBCs characterized by negativity for estrogen receptor (ER), progesterone receptor (PR), and *HER-2* genes, represent 10%–24% of invasive breast cancers. They consist of high-grade tumors with different histologies. Patients with TNBC tend to have a poorer short-term prognosis than other breast cancer types, in part because there are currently no targeted therapies for these tumors [1]. The research of new molecular signatures tailored to this specific subtype therefore represents a fundamental objective [2]. The recent molecular characterization of TNBC [3] revealed the presence of a great heterogeneity, identifying five major classes: (i) “Basal-like” subtype which makes up approximately 25% to 80% of TNBC cases, characterized by biological pathways involving cell cycle and DNA damage response (e.g., ATR/BRCA, etc.); (ii) “Mesenchymal” subtype characterized by genes involved in EMT (epithelial-mesenchymal transition) and in the biological regulation of cancer stem cells; (iii) “Immunomodulatory” subtype enriched in gene ontologies of the immune cell process including immune cell signaling (B, T, and NK cells) and cytokine signaling; (iv) “Luminal AR” subtype enriched in hormonally regulated pathways by AR overexpression; and (v) “*HER2* enriched” subtype which makes up approximately 6% to 8% of TNBC cases, characterized by immuno-positivity for HER2 receptor (IHC score 1+ and 2+) but no gene amplification.

PD-L1 is a transmembrane protein of 40 kDa, expressed on epithelial cells, vascular endothelial cells, natural killer cells, macrophages, myeloid dendritic cells, and B cells [4]. *PD-1/PD-L1* pathway may have a key role in a mechanism of adaptive immune resistance in cancer. Several studies reported an aberrant *PD-L1* expression in many tumors, often correlated with a poor prognosis, suggesting its potential role as prognostic and predictive biomarker [5].

In TNBC, *PD-L1* expression could be associated with “immuno-modulatory” molecular subtype, but its staining and relation with clinic-pathological features and survival have not yet been clearly defined.

Several papers described *PD-L1* expression in BC subtypes displaying data often discordant. In a recent study, IHC PD-L1 expression in a large case series of BC samples was evaluated, highlighting that its expression was significantly associated with age, tumor size, lymph node status and worse OS [6].

In other studies, several authors have considered both stromal and cytoplasmic positivity for PD-L1. Cytoplasmic positivity of PD-L1 was associated with a lower risk of breast cancer death [7]. Moreover, no correlation was made between the expression of PD-L1, clinical-pathological features and outcome of TNBC patients. More recently, another study highlighted that stromal expression of PD-L1 is associated with better Disease-Free Survival in TNBC [8]. In all studies, the variability of the PD-L1 expression in BC can still be strongly influenced by the different antibodies clones used [9].

Finally, the relationships between PD-L1 expression in tumor microenvironment, in particular in TIL cells, and breast cancer cells, was recently investigated, showing no association of TIL PD-L1 expression with clinical-pathological parameters and overall survival [10].

To better define the prognostic role of PD-L1 in TNBC cells and the relation with other clinic-pathologic features, including metabolic profile, we selected a large case series of TNBCs to optimize, by immunohistochemistry, PD-L1 expression on tumor and TIL cells using one of antibody clones approved for diagnostic assay [11].

Our data highlighted that PD-L1 staining in tumor cells are strongly associated with a better disease free survival in TNBCs patients and that its overexpression can be also associated with diabetic disease.

## 2. Results

### 2.1. Clinical-Pathological Characteristics and Follow Up Data of Triple Negative Breast Cancers (TNBC) Patients

In our cohort, we have included 238 TNBC samples. The age of patients ranged 24–93 years, with an average age of 57 years. The percentages of tumor grading were: 88.5% grade III and 11.4% grade I–II. Tumor sizes were: lower than 2 cm in 46.19% of the samples, between 2 and 5 cm in 45.23% (69/155) and larger than 5 cm in 8.5%. Metastatic lymph nodes (LNM) were found in 81.5% of patients while distant metastases were found in 74.1% of cases. Forty cases were unable to recover this information. The expression of proliferation factor Ki67 was high (>20%) in 82.5%, and low (≤20%) in 17.45% of cases. Regarding metabolic parameters, BMI evaluation was ≥30 in 35% of cases but this information was lost for 117 patients. In total, 30.1% of TNBC patients were diabetic but this information was lost for 144 patients. The information about follow up was lost for 135 (56.7%) of patients. All information is schematized in Table 1 and Table S1.

**Figure 1:**
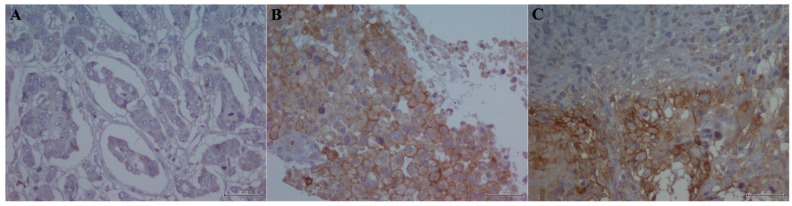
PD-L1 immunostaining in TNBC cells: (**A**) “Score 0”, detail of absence of membranous immunoreactivity (magnification 40×); (**B**) “Score 1”, detail of incomplete membranous positivity with basolateral and/or with semicircular bars (magnification 40×); and (**C**) “Score 2”, detail of complete membranous positivity (magnification 40×).

Regarding stratification of PD-L1 positive samples, 20.3% showed a score 0 (Figure 1A), 15.6% a score 2 (Figure 1B) and 57.4% a score 2 (Figure 1C).

**Figure 2:**
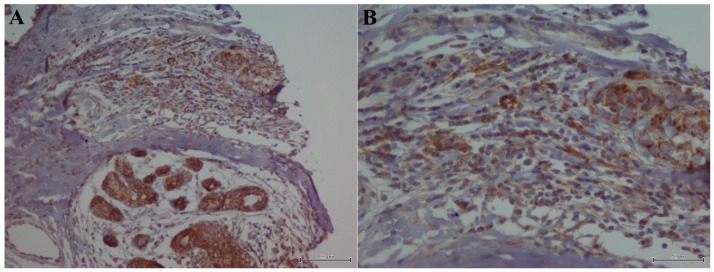
PD-L1 immunostaining in TIL: (**A**) TIL PD-L1+ cells (magnification 20×); and (**B**) detail of positive PD-L1 staining on TIL and tumor cells (magnification 40×).

Regarding PD-L1 staining on TIL cells, only 119 samples were evaluable. We detected its expression in 51 specimens (42.8%) samples (Figure 2A), while combined PD-L1 expression on tumor and TIL cells (Figure 2B) was detected in 35 samples (29.6%) (Table S1).

### 2.2. Association of the PD-L1 Expression with Clinical-Pathological Data

For all statistical elaborations realized, not observing significant differences between samples with different tumor scores, we have only considered the positivity and negativity of PD-L1 tumor staining.

The relation of PD-L1 tumor expression with the clinical-pathological parameters in TNBC showed a direct statistically significant association (*p* ≤ 0.05) between the expression of PD-L1 and the proliferation index (*p* = 0.009). The more significant inverse statistical association was present between PD-L1 expression and menopausal status (*p* = 0.006), lymph nodal metastases (*p* = 0.026) and relapse (*p* = 0.008) (Table 1).

Regarding the relation of PD-L1 TIL expression with the clinical-pathological parameters, we showed only a direct statistically significant association with relapse (*p* = 0.017), and a trend of association with proliferation index Ki67 (*p* = 0.087) (Table S1).

Combined expression of PD-L1 on tumor and TIL cells showed no statistical association with the clinical-pathological parameters (Table S1).

### 2.3. PD-L1 Relation with Metabolic Features of TNBC Patients

Based on statistical elaboration of PD-L1 tumor expression with metabolic parameters in TNBC patients, we showed that no statistical association was present with BMI, while a significant relation with la glycemia (*p* = 0.043) and diabetic disease (*p* = 0.034) was present (Table 1).

PD-L1 TIL expression had a trend of inverse association with glycemia (*p* = 0.079) and diabetes (*p* = 0.088), but this relation was lost if we considered combined expression of PD-L1 on tumor and TIL cells. No statistical association was present with BMI (Table S1).

### 2.4. PD-L1 Expression Association with Survival of TNBC Patients

**Figure 3:**
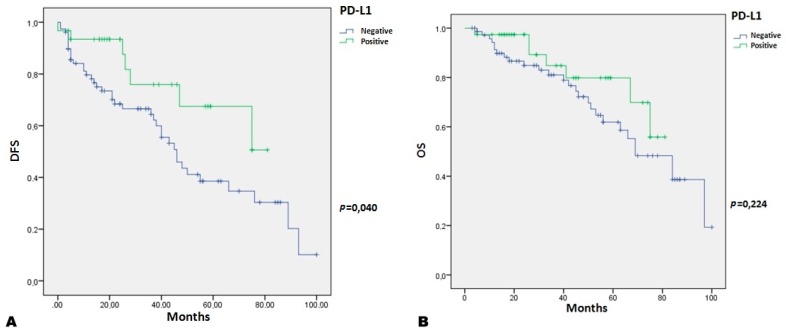
Tumor PD-L1 + TNBC patients Kaplan–Meier curves: (**A**) Disease Free Survival (DFS) (*p* = 0.040); and (**B**) Overall Survival (OS) (*p* = 0.224).

Regarding the relation with patient survival, we showed that tumor PD-L1 expression is strongly associated with better disease-free survival (DFS). A strong statistical association between PD-L1 + TNBC and DFS was present (*p* = 0.040) (Figure 3A). However, the statistical association with overall survival (OS) are not significant (*p* = 0.224) (Figure 3B).

No statistical association was present between TIL PD-L1 staining, combined expression of tumor and TIL PD-L1 expression and TNBC patient survival (Figures S1 and S2).

## 3. Discussion

In this study, we aimed to analyze the expression of PD-L1 on tumor and TIL cells of TNBC cases to determine the real prognostic value of PD-L1 in these lesions, optimizing immunohistochemistry method, using one of the clones of antibodies approved for diagnostic assay [11].

TNBCs appear resistant to conventional therapeutic regimens. Their incidence is particularly high in younger women and the clinical course of the disease is often very aggressive compared with other breast cancer subtypes [2,12,13]. Chemotherapy is currently the mainstay of systemic treatment for these patients. This explains the growing interest from the clinical research for this tumor subtype and, above all, the need to identify new molecular prognostic and predictive markers. Numerous studies have shown that the PD-1/PD-L1 pathway plays a key role in the interaction between tumor cells and cells responsible for immune response, and its presence on the tumor cell surface can function as an immune resistance mechanism (“tumor escape”) [14].

In recent years, the use of drugs for immune checkpoint control has revolutionized the scenario of traditional treatment regimens [15]. In particular, the advent and success of the PD-1/PD-L1 pathway inhibitors, administered as single agents or in combination with other drugs, has led to the hypothesis that PD-L1 detection on the surface of tumor cells could be a useful tool in the stratification of patients that can benefit from these therapies [16].

Many solid tumors express PD-L1 on their surface, but to this expression has been assigned a prognostic significance often contrasting. This may be due to the different antibody clones used for its immunohistochemical identification, variability in the different tumor types, and an inadequately defined evaluation score [12].

In addition, for breast cancers, the expression data described in the literature are highly variable in different tumor subtypes. Soliman et al. analyzed PD-L1 expression in cell models of breast cancer showing its overexpression in particular in the basal phenotype [17]. Sabatier and collaborators have analyzed the expression of PD-L1 in 45 cell lines and on a large series of breast cancer (5454) using a DNA microarray, pointing out that its up-regulation is associated with a better prognosis and response to chemotherapy [18].

However, in another recent study, the immunohistochemical expression of PD-L1 was analyzed in a series of 650 breast cancers showing a statistically significant association with age, tumor size, lymph node status and worse overall survival [6]. In this study, immunohistochemical analysis results were not sufficiently clear, showing a predominant cytoplasmic positivity instead of physiological membrane positivity associated with the receptor.

Several studies described also the importance of TIL PD-L1 expression detection, and recently it was shown that its expression on immune cells, but not on tumor cells, is a favorable prognostic factor for head and neck cancer patients [19].

Our results showed a differential expression of the biomarker on TNBC cells, suggesting the need of adequate evaluation score, and on TIL cells in tumor microenvironment.

Whereas TIL PD-L1 staining showed no statistically significant association with clinical-pathological features and patients survival, in line with what already described in the literature for other BC subtypes [10], we showed a direct statistically significant association between the expression of tumor PD-L1 and the proliferation index, and an inverse correlation between the expression of the protein and menopause, the presence of lymph node metastasis and recurrence. Moreover, the tumor expression of PD-L1 was strongly associated with a better disease-free survival (DFS), but not correlated with OS according with other studies realized on breast cancer [20].

From our results, a statistically significant association of tumor PD-L1 expression with metabolic disorders such as diabetes also emerged. It is known that the metabolic diseases contribute to determine a predisposition to some neoplastic diseases [21,22]. In particular, the role of prediabetes and diabetes in increasing breast cancer risk has been abundantly documented in recent studies [23]. However, Reeves et al. showed that diabetes was associated with a significant, albeit borderline, increased risk of ER+ and PR+ breast cancer but not with ER- or PR-breast cancer [24]. In fact, in our case series the presence of diabetic disease would seem even related to a better prognosis. It is unclear, however, whether, in patients with overt diabetes, using anti-diabetic drugs may contribute in part to improve the prognosis. Recently, it has been shown that certain anti-diabetic drugs, as metformin, may have anti-tumor activity, significantly contributing to improve the outcome of patients [25]. This positive effect may be enhanced in TNBC patients, because it was recently shown that in particular ERalpha receptor plays an important role in hyperglycemia-induced chemo-resistance, only observed in ERα+ breast cancer cells [26].

However, the biological mechanisms behind a potential increased risk of breast cancer in women with diabetes are not well understood, therefore our experimental data can only contribute to speculate.

## 4. Materials and Methods

### 4.1. Patients and Specimens

From 2003 to 2013, 238 patients who underwent a mastectomy, quadrantectomy or metastectomy at the National Cancer Institute “Giovanni Pascale Foundation” of Naples, Italy, were enrolled into this study. All subjects gave their informed consent for inclusion before they participated in the study. The study was conducted in accordance with the Declaration of Helsinki, and the protocol was approved by the Ethics Committee of INT “Giovanni Pascale Foundation” (Project M2/4).

In our institution, the percentage of tumors classified as Triple Negative is approximately 15%–19% of the total number of breast cancer surgical samples. All cases of Triple Negative and non-Triple Negative breast samples were reviewed according to WHO classification criteria, using standard tissue sections and appropriate immunohistochemical slides.

Medical records for all cases of Triple Negative breast samples were reviewed for clinical information, including histologic parameters that were determined from the H&E slides. The following clinical and pathological parameters were evaluated for each tumor included in the study: patient age at initial diagnosis, tumor size, histologic subtype, histologic grade, nuclear grade, nodal status, number of positive lymph nodes, tumor stage, tumor recurrence or distant metastasis and type of surgery (for tumor removal).

In addition, all specimens were characterized for all routinely diagnostic immunophenotypic parameters.

### 4.2. TMA Building

Tissue Micro-Array (TMA) was built using the most representative areas from each single case with one replicate. All tumors and controls were reviewed by two experienced pathologists (MDB/GB). Discrepancies between two pathologists from the same case were resolved in a joint analysis of the cases. Tissue cylinders with a diameter of 0.6 mm were punched from morphologically representative tissue areas (cores of intra-tumoral and cores of peri-tumoral tissues) of each “donor” tissue block and brought into one recipient paraffin block (3 cm × 2.5 cm) using a semi-automated tissue arrayer (Galileo TMA) as previously described [27,28].

### 4.3. Immunohistochemistry Analysis

Immunohistochemical staining was done on slides from formalin-fixed, paraffin embedded tissues to evaluate the expression of ER PgR, HER2, Ki67 and PD-L1 markers. Paraffin slides were de-paraffinized in xylene and rehydrated through graded alcohols. Antigen retrieval was performed with slides heated in 0.01 M citrate buffer (pH 6.0) in a bath for 20 min at 97 °C. After antigen retrieval, the slides allow to cool. The slides were rinsed with TBS and the endogenous peroxidase was inactivated with 3% hydrogen peroxide. After protein block (BSA 5% in PBS 1×), the slides were incubated with primary antibody to human to human ERα (Monoclonal Mouse Anti-Human Erα, Clone ID5, dilution 1:35, Dako North America, Inc., Carpinteria, CA, USA), PR (Monoclonal Mouse Anti-Human PR, Clone 636, dilution 1:50, Dako North America, Inc.), c-Erb B2 (Polyclonal Rabbit Anti-Human Oncoprotein, dilution 1:300, Dako North America, Inc.), Ki67 (Monoclonal Mouse Anti-Human Ki67 Ag Clone MIB-1, dilution 1:75, Dako North America, Inc.) for 30 min. Immunohistochemistry protocol for PD-L1 staining was previously optimized [28] with SP142 antibody clone (Spring bioscience (M4420) Koll Center Pkwy, Pleasanton, CA, USA) at a concentration of 3.75 µg/mL, incubated for 90 min.

The sections were rinsed in TBS and incubated for 20 min with Biotinylated Secondary Antibody (RE7103, Novocastra, Leica, Wetzlar, Germany), a biotin-conjugated secondary antibody formulation that recognized mouse and rabbit immunoglobulins. Then, the sections were rinsed in TBS and incubated for 20 min with Streptavidin-HRP (RE7104, Novocastra). For PD-L1, the secondary antibody was incubate for 40 min, followed by incubation with DAB substrate buffer + DAB chromogen (3,3′-diaminobenzidine) (SK001, DAKO, Glostrup, Denmark). Between all incubation steps, slides were extensively washed with Bond Wash Solution (SK310, DAKO). Finally, peroxidase reactivity was visualized using a 3,3′-diaminobenzidine (DAB) and the sections were counterstained with hematoxylin and mounted. Results are interpreted using a light microscope (Olympus BX51, Tokyo, Japan).

### 4.4. Evaluation of Immunohistochemistry on TIL and Tumor Cells

Antigen expression was evaluated independently by a pathologist using a light microscopy. Observer was unaware of the clinical outcome. For each sample, at least five fields (inside the tumor and in the area exhibiting tumor invasion) (Figure S3) and >500 cells were analyzed. Using a semi-quantitative scoring system microscopically and referring to each antigen scoring method in other studies, an observer evaluated the intensity, extent and subcellular distribution.

For TIL characterization, lymphocytes were quantified by manual expression scoring, considering as tumor infiltrating (TIL) if they were located within 1 high-power field (HPF; ×40) of carcinoma cells. The total number of TIL/HPF was counted in each TMA core and averaged across the case to give the mean number of TIL/HPF. An immunohistochemistry characterization of immune cells was performed by CD3 (for T-cells), CD20 (for B-cells) [29] (Figure S4).

The evaluation criteria used for ER, PR, c-ErbB-2 and proliferative index Ki67 tumor expression are previously described [30].

For tumor PD-L1 assessment and a score definition, we considered both a qualitative and a quantitative parameter. For the qualitative criteria we considered the immunoreactivity of membrane dividing it into “absent”, “incomplete” and “complete”, and the intensity of the reaction at the membrane level, dividing it into “mild”, “moderate” and “intense”. For the quantitative criteria, we considered the percentage of positive tumor cells ≥10%. We defined as “score 0” cases with the absence of membranous immunoreactivity or mild/moderate cytoplasmic positivity; “Score 1+” cases with incomplete membranous positivity, which can be the basolateral and/or with semicircular bars, with a moderate/intense immunoreactivity, with/without cytoplasmic positivity, in ≥10% of tumor cells; “Score 2+” cases with complete membranous positivity, with a moderate/intense immunoreactivity, with/without cytoplasmic positivity (Figure S5), in ≥10% of tumor cells.

For TIL PD-L1 staining, we considered only the percentage of positive cells, defining negative/low the staining in ≤10% of cells and positive/high in ≥10% of cells.

### 4.5. Statistical Analysis

The association between PD-L1 expression with clinical-pathological parameters and was conducted using the χ^2^ and *t*-student test.

The Pearson χ^2^ test was used to determine whether a relationship exists between the variables included in the study. The level of significance was defined as *p* < 0.05. Overall survival (OS) and disease-free survival (DFS) curves were calculated using the Kaplan–Meier method. All the statistical analyses were carried out using the Statistical Package for Social Science v. 20 software (SPSS Inc., Chicago, IL, USA).

OS was defined as the time from diagnosis (first biopsy) to death by any cause or until the most recent follow-up. DFS was measured as the time from diagnosis to the occurrence of progression, relapse after complete remission, or death from any cause. DFS had a value of zero for patients who did not achieve complete remission. The follow-up duration was five years.

## 5. Conclusions

In conclusion, our data allowed clarifying the real prognostic value of PD-L1, identifying a category of patients that, basing on the tumor cells expression, could be enrolled for the use of inhibitory immune checkpoint therapies. In addition, its association with diabetes could once again mean contemplating the use of combined biological therapies for these patients.

## Figures and Tables

**Table 1:** Statistical associations between PD-L1 tumor expression and clinical pathological features of Triple Negative Breast Cancers (TNBC) samples. (*) indicates statistical significance.

Clinical Pathological Features	TUMOR PD-L1		*p*-Value	R Pearson
	Low	High		
**Age**				
<40	15 (60%)	10 (40%)	0.129	−0.118
≥40 and ≤60	56 (58.3%)	40 (41.7%)		
>60	69 (71.9%)	27 (28.1%)		
**Histotype**				
Ductal	120 (62.9)	71 (37.1)	0.103	−0.110
Non Ductal	22 (78.6)	6 (21.4)		
**Menopausal State**				
Pre	41 (52.6%)	37 (47.4)	0.006 *	−0.187
Post	99 (71.2%)	40 (28.8%)		
**Tumor Size (cm)**				
≤2	63 (64.9%)	34 (35.1%)	0.769	−0.018
>2 and ≤5	60 (63.2%)	35 (36.8%)		
>5	13 (72.2%)	5 (27.8%)		
**LNM**				
Negative	61 (60.4%)	40 (39.6%)	0.026 *	−0.168
Positive	58 (76.3%)	18 (23.7%)		
**Metastases**				
Negative	82 (66.7%)	41 (33.3%)	0.079	−0.138
Positive	31 (81.6%)	7 (18.4%)		
**Grade**				
G1	2 (100%)	0 (0%)	0.494	0.068
G2	15 (68.2%)	7 (31.8%)		
G3	116 (62.7%)	69 (37.3%)		
**Ki-67**				
Low	31 (83.8%)	6 (16.2%)	0.009 *	0.180
High	107 (61.1%)	68 (38.9%)		
**BMI**				
<30	48 (73.8%)	17 (26.2%)	0.962	−0.005
≥30	26 (74.3%)	9 (25.7%)		
**Diabetes**				
No	42 (82.4%)	9 (17.6%)	0.034 *	0.248
Yes	13 (59.1%)	9 (40.9)		
**Glicemya**				
<110	43 (82.7%)	9 (17.3%)	0.043 *	0.244
>110	10 (58.8%)	7 (41.2%)		
**Status**				
Alive	66 (66.7%)	33 (32.3%)	1	0.000
Dead	10 (66.7%)	5 (32.3%)		
**Relapse**				
No	47 (61.8%)	29 (38.2)	0.008 *	−0.238
Yes	42 (84%)	8 (16%)		

## Supplementary Materials

Supplementary materials can be found at www.mdpi.com/1422-0067/18/2/459/s1.
