# Supplementary material for: Programmed Death Ligand 1 (PD-L1) Tumor Expression Is Associated with a Better Prognosis and Diabetic Disease in Triple Negative Breast Cancer Patients
Source: Int J Mol Sci. 2017 Feb 21;18(2):459. doi: 10.3390/ijms18020459 (PMC5343992; doi:10.3390/ijms18020459)
Supplement: Supplementary file 1 [file ijms-18-00459-s001.pptx]

## Slide 1
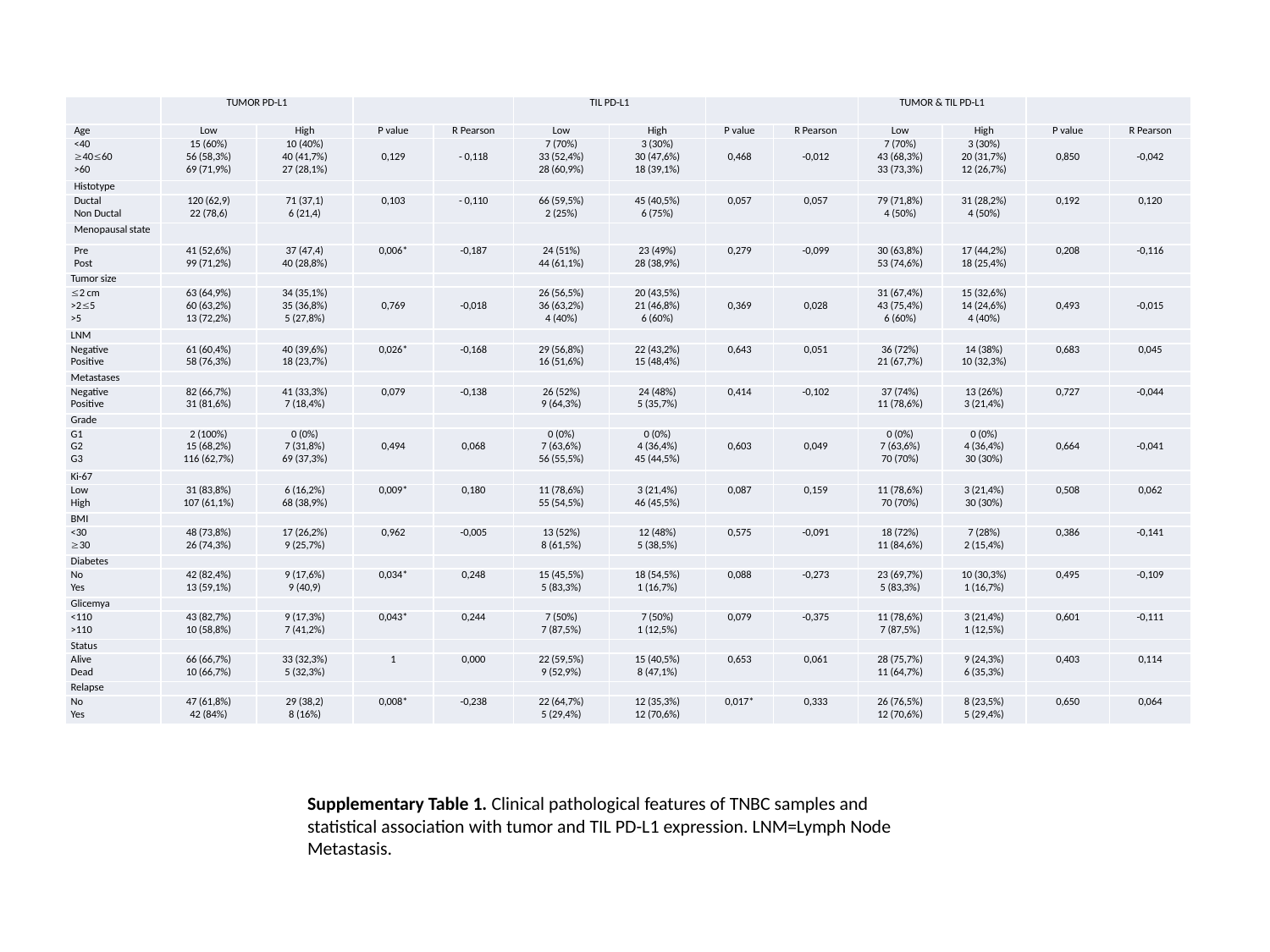

| | TUMOR PD-L1 | | | | TIL PD-L1 | | | | TUMOR & TIL PD-L1 | | | |
| --- | --- | --- | --- | --- | --- | --- | --- | --- | --- | --- | --- | --- |
| Age | Low | High | P value | R Pearson | Low | High | P value | R Pearson | Low | High | P value | R Pearson |
| <40 ≥4060 >60 | 15 (60%) 56 (58,3%) 69 (71,9%) | 10 (40%) 40 (41,7%) 27 (28,1%) | 0,129 | - 0,118 | 7 (70%) 33 (52,4%) 28 (60,9%) | 3 (30%) 30 (47,6%) 18 (39,1%) | 0,468 | -0,012 | 7 (70%) 43 (68,3%) 33 (73,3%) | 3 (30%) 20 (31,7%) 12 (26,7%) | 0,850 | -0,042 |
| Histotype | | | | | | | | | | | | |
| Ductal Non Ductal | 120 (62,9) 22 (78,6) | 71 (37,1) 6 (21,4) | 0,103 | - 0,110 | 66 (59,5%) 2 (25%) | 45 (40,5%) 6 (75%) | 0,057 | 0,057 | 79 (71,8%) 4 (50%) | 31 (28,2%) 4 (50%) | 0,192 | 0,120 |
| Menopausal state | | | | | | | | | | | | |
| Pre Post | 41 (52,6%) 99 (71,2%) | 37 (47,4) 40 (28,8%) | 0,006\* | -0,187 | 24 (51%) 44 (61,1%) | 23 (49%) 28 (38,9%) | 0,279 | -0,099 | 30 (63,8%) 53 (74,6%) | 17 (44,2%) 18 (25,4%) | 0,208 | -0,116 |
| Tumor size | | | | | | | | | | | | |
| 2 cm >25 >5 | 63 (64,9%) 60 (63,2%) 13 (72,2%) | 34 (35,1%) 35 (36,8%) 5 (27,8%) | 0,769 | -0,018 | 26 (56,5%) 36 (63,2%) 4 (40%) | 20 (43,5%) 21 (46,8%) 6 (60%) | 0,369 | 0,028 | 31 (67,4%) 43 (75,4%) 6 (60%) | 15 (32,6%) 14 (24,6%) 4 (40%) | 0,493 | -0,015 |
| LNM | | | | | | | | | | | | |
| Negative Positive | 61 (60,4%) 58 (76,3%) | 40 (39,6%) 18 (23,7%) | 0,026\* | -0,168 | 29 (56,8%) 16 (51,6%) | 22 (43,2%) 15 (48,4%) | 0,643 | 0,051 | 36 (72%) 21 (67,7%) | 14 (38%) 10 (32,3%) | 0,683 | 0,045 |
| Metastases | | | | | | | | | | | | |
| Negative Positive | 82 (66,7%) 31 (81,6%) | 41 (33,3%) 7 (18,4%) | 0,079 | -0,138 | 26 (52%) 9 (64,3%) | 24 (48%) 5 (35,7%) | 0,414 | -0,102 | 37 (74%) 11 (78,6%) | 13 (26%) 3 (21,4%) | 0,727 | -0,044 |
| Grade | | | | | | | | | | | | |
| G1 G2 G3 | 2 (100%) 15 (68,2%) 116 (62,7%) | 0 (0%) 7 (31,8%) 69 (37,3%) | 0,494 | 0,068 | 0 (0%) 7 (63,6%) 56 (55,5%) | 0 (0%) 4 (36,4%) 45 (44,5%) | 0,603 | 0,049 | 0 (0%) 7 (63,6%) 70 (70%) | 0 (0%) 4 (36,4%) 30 (30%) | 0,664 | -0,041 |
| Ki-67 | | | | | | | | | | | | |
| Low High | 31 (83,8%) 107 (61,1%) | 6 (16,2%) 68 (38,9%) | 0,009\* | 0,180 | 11 (78,6%) 55 (54,5%) | 3 (21,4%) 46 (45,5%) | 0,087 | 0,159 | 11 (78,6%) 70 (70%) | 3 (21,4%) 30 (30%) | 0,508 | 0,062 |
| BMI | | | | | | | | | | | | |
| <30 ≥30 | 48 (73,8%) 26 (74,3%) | 17 (26,2%) 9 (25,7%) | 0,962 | -0,005 | 13 (52%) 8 (61,5%) | 12 (48%) 5 (38,5%) | 0,575 | -0,091 | 18 (72%) 11 (84,6%) | 7 (28%) 2 (15,4%) | 0,386 | -0,141 |
| Diabetes | | | | | | | | | | | | |
| No Yes | 42 (82,4%) 13 (59,1%) | 9 (17,6%) 9 (40,9) | 0,034\* | 0,248 | 15 (45,5%) 5 (83,3%) | 18 (54,5%) 1 (16,7%) | 0,088 | -0,273 | 23 (69,7%) 5 (83,3%) | 10 (30,3%) 1 (16,7%) | 0,495 | -0,109 |
| Glicemya | | | | | | | | | | | | |
| <110 >110 | 43 (82,7%) 10 (58,8%) | 9 (17,3%) 7 (41,2%) | 0,043\* | 0,244 | 7 (50%) 7 (87,5%) | 7 (50%) 1 (12,5%) | 0,079 | -0,375 | 11 (78,6%) 7 (87,5%) | 3 (21,4%) 1 (12,5%) | 0,601 | -0,111 |
| Status | | | | | | | | | | | | |
| Alive Dead | 66 (66,7%) 10 (66,7%) | 33 (32,3%) 5 (32,3%) | 1 | 0,000 | 22 (59,5%) 9 (52,9%) | 15 (40,5%) 8 (47,1%) | 0,653 | 0,061 | 28 (75,7%) 11 (64,7%) | 9 (24,3%) 6 (35,3%) | 0,403 | 0,114 |
| Relapse | | | | | | | | | | | | |
| No Yes | 47 (61,8%) 42 (84%) | 29 (38,2) 8 (16%) | 0,008\* | -0,238 | 22 (64,7%) 5 (29,4%) | 12 (35,3%) 12 (70,6%) | 0,017\* | 0,333 | 26 (76,5%) 12 (70,6%) | 8 (23,5%) 5 (29,4%) | 0,650 | 0,064 |
Supplementary Table 1. Clinical pathological features of TNBC samples and statistical association with tumor and TIL PD-L1 expression. LNM=Lymph Node Metastasis.

## Slide 2
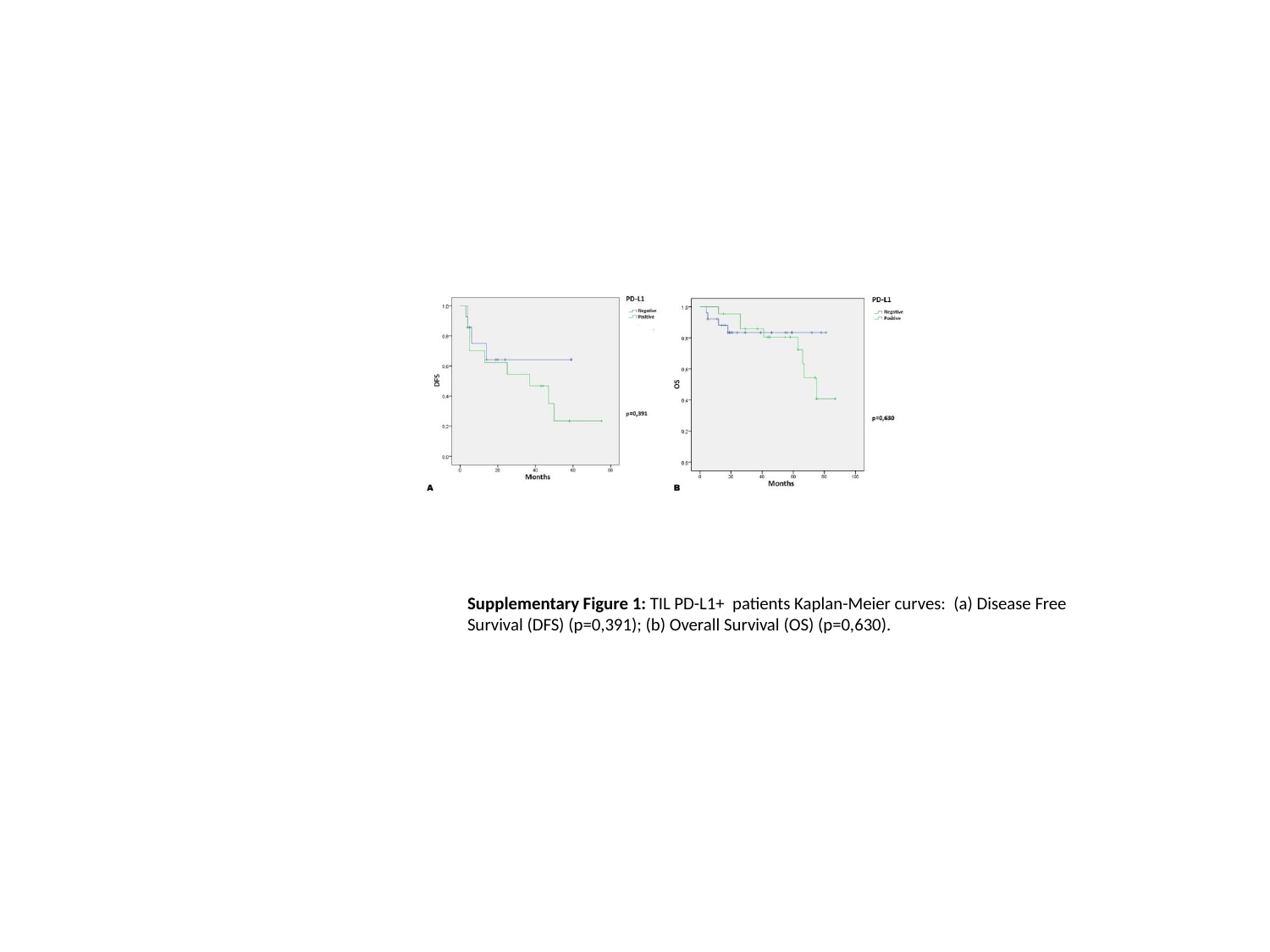

Supplementary Figure 1: TIL PD-L1+ patients Kaplan-Meier curves: (a) Disease Free Survival (DFS) (p=0,391); (b) Overall Survival (OS) (p=0,630).

## Slide 3
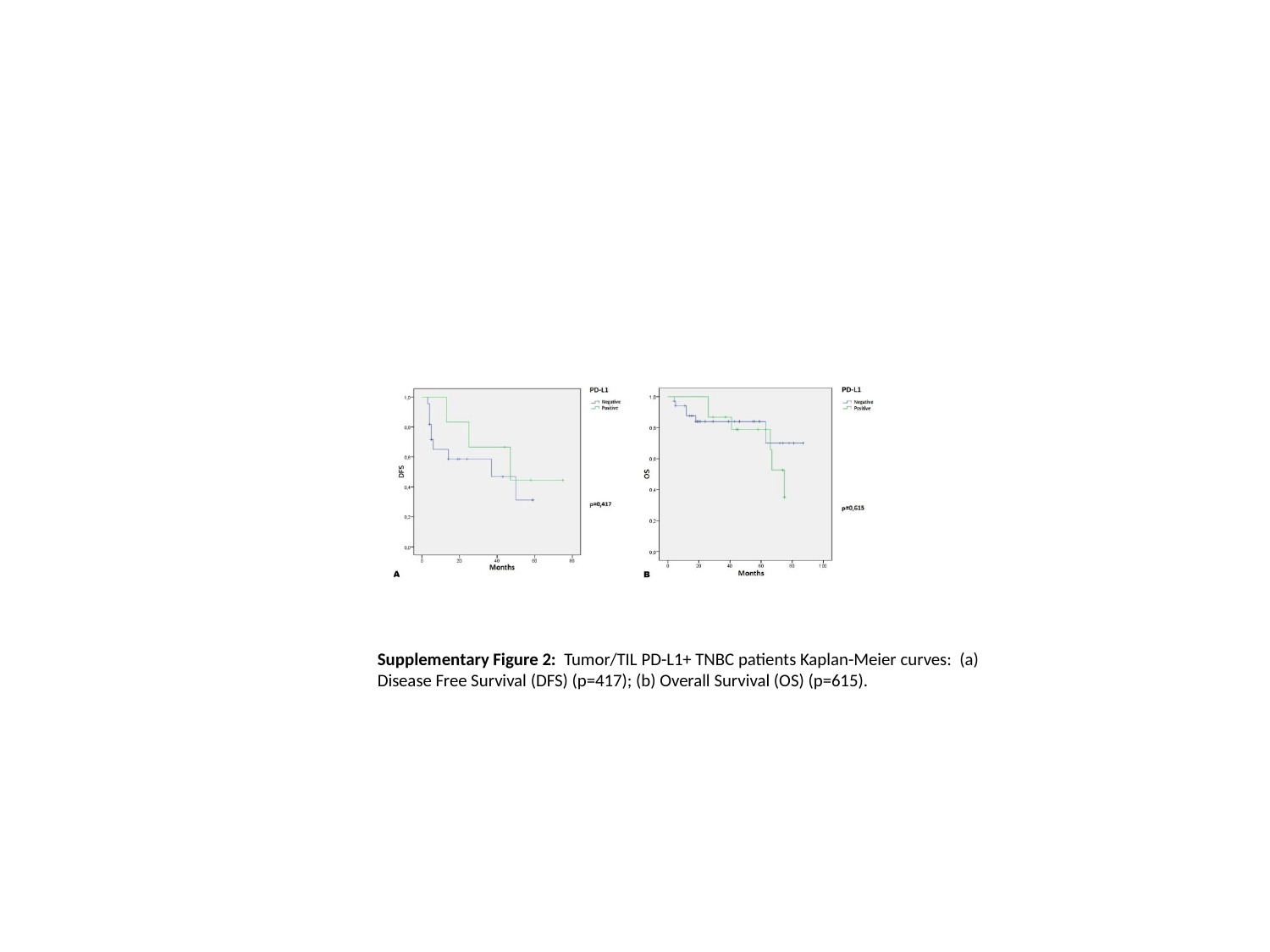

Supplementary Figure 2: Tumor/TIL PD-L1+ TNBC patients Kaplan-Meier curves: (a) Disease Free Survival (DFS) (p=417); (b) Overall Survival (OS) (p=615).

## Slide 4
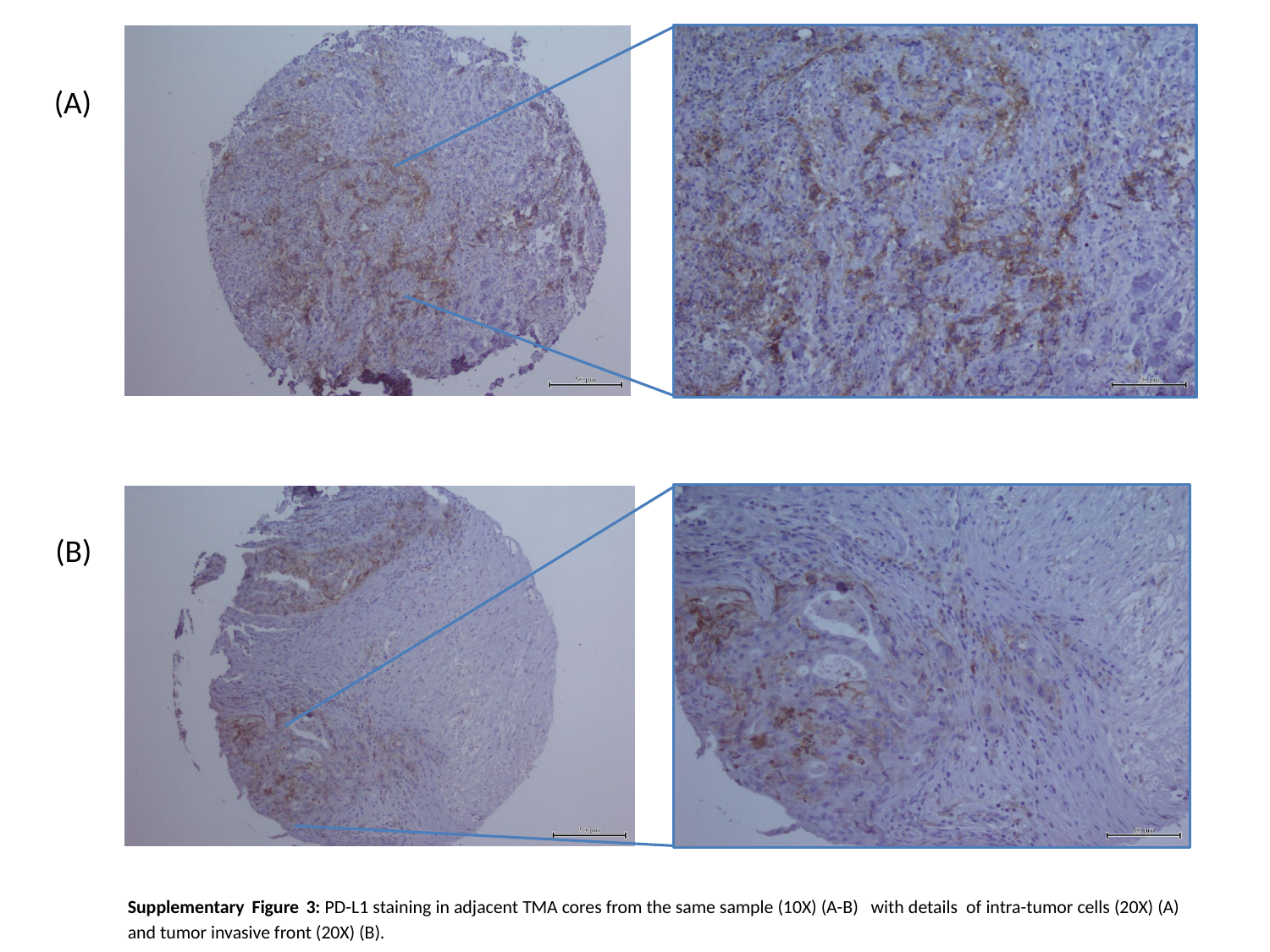

(A)
(B)
Supplementary Figure 3: PD-L1 staining in adjacent TMA cores from the same sample (10X) (A-B) with details of intra-tumor cells (20X) (A) and tumor invasive front (20X) (B).

## Slide 5
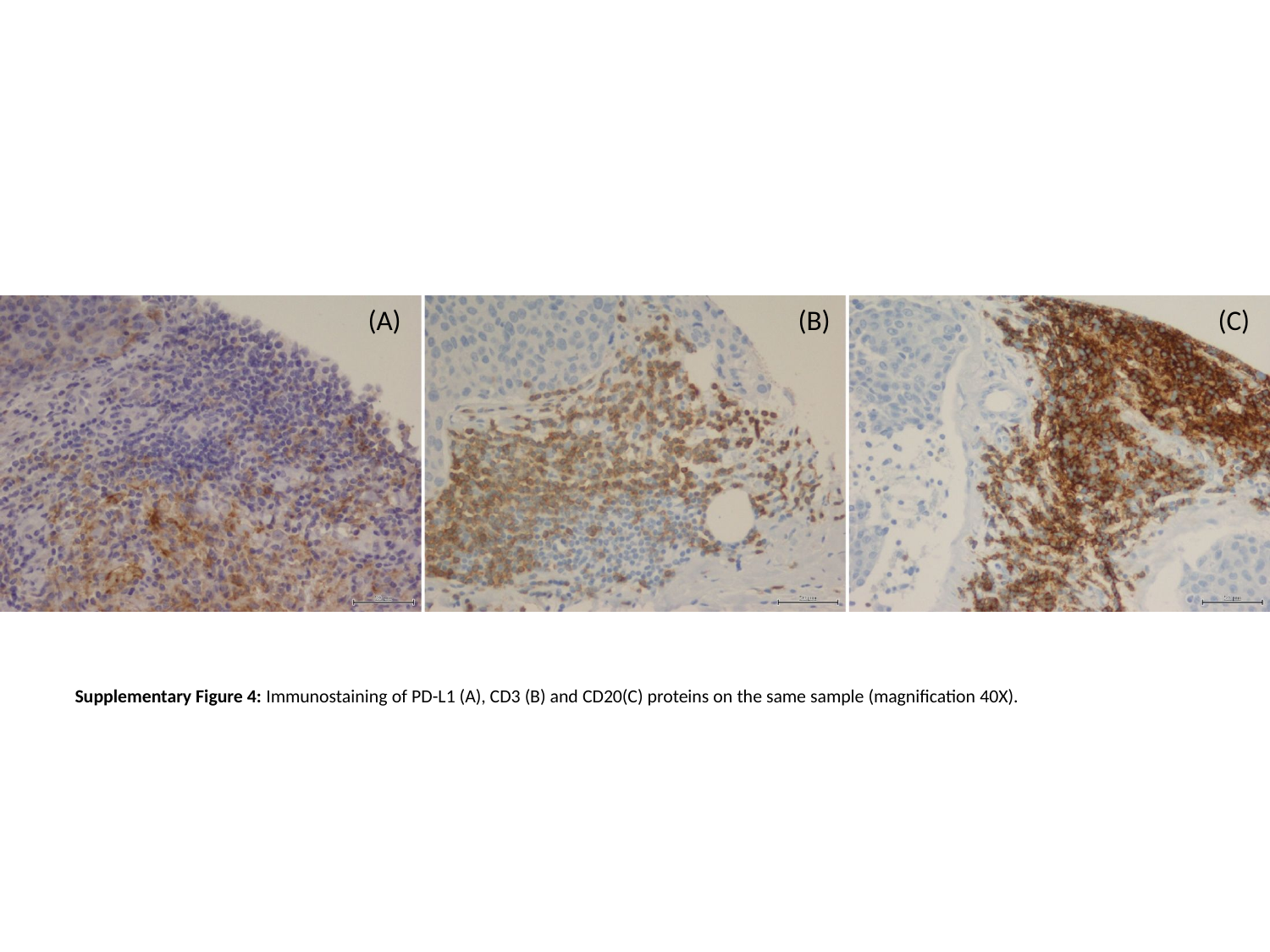

(A)
(B)
(C)
Supplementary Figure 4: Immunostaining of PD-L1 (A), CD3 (B) and CD20(C) proteins on the same sample (magnification 40X).

## Slide 6
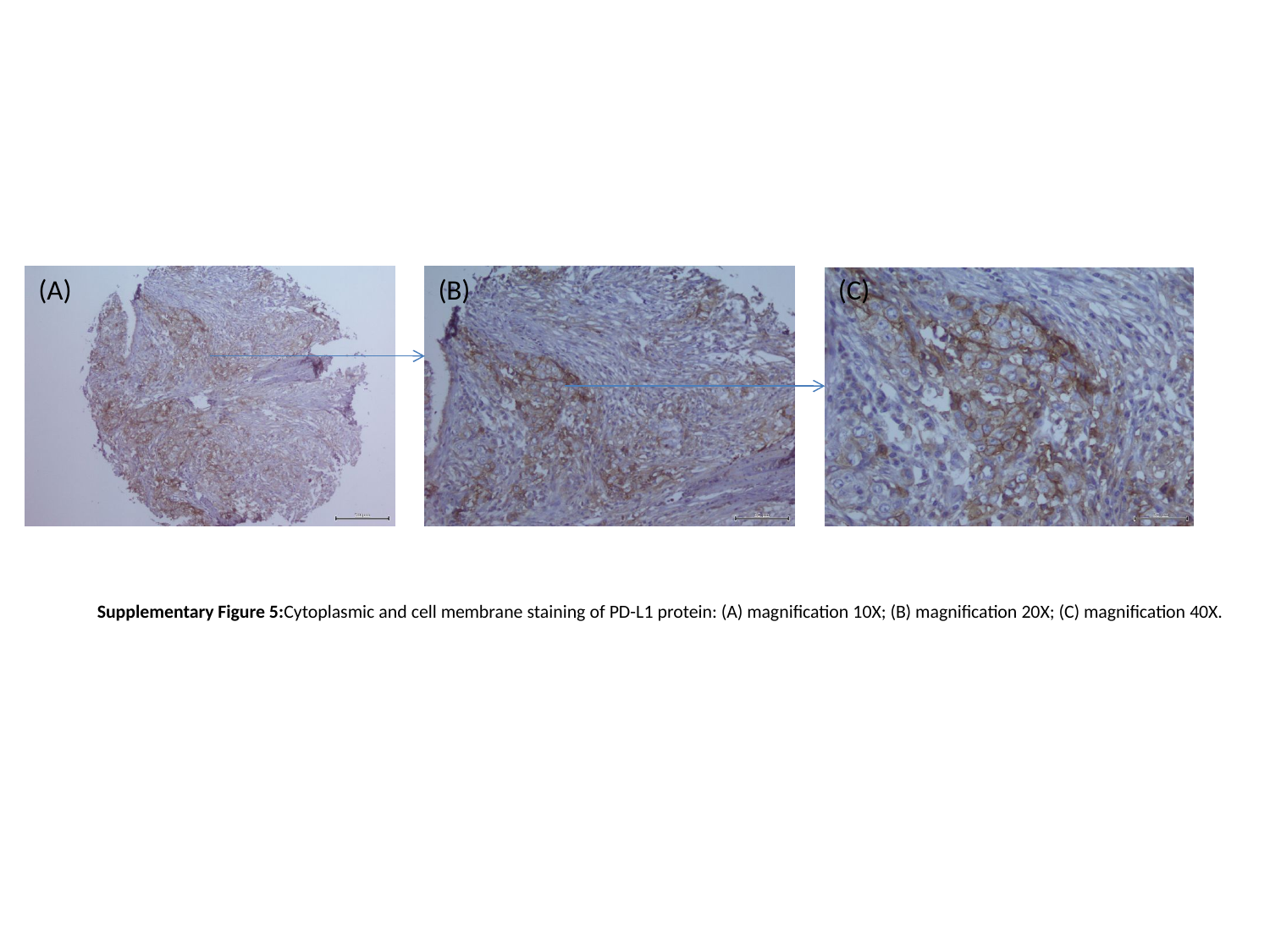

(A)
(B)
(C)
Supplementary Figure 5:Cytoplasmic and cell membrane staining of PD-L1 protein: (A) magnification 10X; (B) magnification 20X; (C) magnification 40X.
